# A nested real-time PCR assay for the quantification of *Plasmodium falciparum* DNA extracted from dried blood spots

**DOI:** 10.1186/1475-2875-13-393

**Published:** 2014-10-04

**Authors:** Tuan M Tran, Amirali Aghili, Shanping Li, Aissata Ongoiba, Kassoum Kayentao, Safiatou Doumbo, Boubacar Traore, Peter D Crompton

**Affiliations:** Laboratory of Immunogenetics, National Institute of Allergy and Infectious Diseases, National Institutes of Health, Twinbrook 2, Room 125, 12441 Parklawn Drive, Rockville, Maryland 20852 USA; Mali International Center of Excellence in Research, University of Sciences, Techniques, and Technology of Bamako (USTTB), BP: 1805 Point G, Bamako, Mali

**Keywords:** *Plasmodium falciparum*, Nucleic acid testing, Quantitative PCR, Nested PCR, Dried blood spot, Passive surveillance

## Abstract

**Background:**

As public health efforts seek to eradicate malaria, there has been an emphasis on eliminating low-density parasite reservoirs in asymptomatic carriers. As such, diagnosing submicroscopic *Plasmodium* infections using PCR-based techniques has become important not only in clinical trials of malaria vaccines and therapeutics, but also in active malaria surveillance campaigns. However, PCR-based quantitative assays that rely on nucleic acid extracted from dried blood spots (DBS) have demonstrated lower sensitivity than assays that use cryopreserved whole blood as source material.

**Methods:**

The density of *Plasmodium falciparum* asexual parasites was quantified using genomic DNA extracted from dried blood spots (DBS) and the sensitivity of two approaches was compared: quantitative real-time PCR (qPCR) targeting the *P. falciparum* 18S ribosomal RNA gene, either with an initial conventional PCR amplification prior to qPCR (nested qPCR), or without an initial amplification (qPCR only). Parasite densities determined by nested qPCR, qPCR only, and light microscopy were compared.

**Results:**

Nested qPCR results in 10-fold higher sensitivity (0.5 parasites/μl) when compared to qPCR only (five parasites/ul). Among microscopy-positive samples, parasite densities calculated by nested qPCR correlated strongly with microscopy for both asymptomatic (Pearson’s r = 0.58, P < 0.001) and symptomatic (Pearson’s r = 0.70, P < 0.0001) *P. falciparum* infections.

**Conclusion:**

Nested qPCR improves the sensitivity for the detection of *P. falciparum* blood-stage infection from clinical DBS samples. This approach may be useful for active malaria surveillance in areas where submicroscopic asymptomatic infections are prevalent.

**Electronic supplementary material:**

The online version of this article (doi:10.1186/1475-2875-13-393) contains supplementary material, which is available to authorized users.

## Background

Although light microscopy examination of peripheral blood smears remains the gold standard for malaria diagnosis and enumerating *Plasmodium* parasites, more sensitive molecular techniques that amplify parasite DNA using polymerase chain reaction (PCR) are routinely applied in clinical studies and epidemiological surveys for the detection and monitoring of low-density, submicroscopic infections [1, 2]. Moreover, sensitive diagnostic assays are becoming increasingly important as malaria eradication efforts seek to eliminate asymptomatic infections that serve as reservoirs for transmission [3, 4].

Quantification of low-density infections allows for determination of parasite growth dynamics in semi-immune individuals [5] or in the context of controlled human malaria infection during clinical vaccine trials [2, 5–7]. Such studies have employed quantitative real-time PCR (qPCR) assays that have a detection limit of 0.02-0.06 parasites per μl but required extraction of DNA from at least 50 μl of cryopreserved whole blood [2, 4, 8], an inconvenient requirement for routine field studies. In contrast, dried filter paper blood spots (DBS) have been a practical way to archive parasite DNA for future analysis without the need for cryopreservation in field settings. Prior studies have shown that quantitative real-time PCR (qPCR) techniques using genomic DNA obtained from DBS samples can reliably detect only ~4 to 40 parasites per μl [9, 10], which is only a modest improvement in sensitivity relative to thick-smear microscopy. Improving the ability to quantify low-density blood-stage infections would be beneficial, especially in studies in endemic areas where asymptomatic infections are common and often submicroscopic. The present study describes an approach that combines nested PCR with qPCR to increase the sensitivity to detect and quantify parasite DNA extracted from clinical DBS samples by at least one order of magnitude over existing qPCR-only methods.

## Methods

### Ethics

Clinical samples used in this study were obtained from an ongoing prospective, cohort study of malaria immunity in Kalifabougou, Mali that began in May 2011. The details of this cohort have been previously described [9]. The Ethics Committee of the Faculty of Medicine, Pharmacy, and Dentistry at the University of Sciences, Techniques, and Technology of Bamako and the Institutional Review Board of the National Institute of Allergy and Infectious Diseases, National Institutes of Health approved this study (NIAID IRB Protocol # 11-I-N126). Written, informed consent was obtained from adult participants and from the parents or guardians of participating children before screening and enrollment.

### Study design and sample collection

Blood samples in this study were obtained during passive and active malaria surveillance visits (occurring every two weeks) in Kalifabougou from May 2011 to December 2011 as previously described [9]. For each participant, peripheral blood was collected by fingerprick for 1) DBS samples archived on 903 Protein Saver filter paper (Whatman) and stored at 25**°**C with silica desiccant in sealed foil envelopes and 2) thick blood smears. Blood smears were stained with Giemsa, and *P. falciparum* parasites were counted against 300 leukocytes. Parasite densities were recorded as the number of asexual parasites/μl of whole blood based on an average leukocyte count of 7500 cells/μL. During symptomatic visits, contemporaneous blood smears were performed for malaria diagnosis and appropriate treatment was initiated per the National Malaria Control Programme guidelines in Mali. Symptoms that initiated a diagnostic evaluation for malaria included fever, chills, sweats, fatigue, headache, nausea, vomiting, and general malaise.

### Sample preparation

Initial screening for *P. falciparum* infections was performed using a non-quantitative, nested PCR technique that detects parasite DNA directly from a 1-mm circular punch of DBS at a sensitivity of ~1 parasite/μl as previously described [9]. For each participant, blood-smear microscopy and non-quantitative, nested PCR were performed on blood samples in chronological order starting from the initial enrolment visit until the first *P. falciparum* infection was detected. For DBS samples identified as *P. falciparum*-positive by this initial screen, genomic DNA (gDNA) was extracted from three 3-mm circular punches containing uniform amounts of blood using the QIAmp DNA Mini kit (Qiagen) per the manufacturer’s protocol with a final elution volume of 150 μl of AE buffer. The extraction protocol was followed rigorously to ensure that the final DNA concentration reflected the maximum obtainable yield. Parasite density calibration standards were generated from 10-fold serial dilutions of purified plasmid DNA containing a single copy of the *P. falciparum* 18S ribosomal RNA (rRNA; MRA-177, MR4, ATCC) [11] at concentrations of 10^9^ copies/μl down to 1 copy/μl diluted in PCR-grade water and gDNA extracted from a DBS sample from a *P. falciparum*-infected patient with a known parasite density of >500,000 parasites/μl as determined by light microscopy at concentrations of ~500,000 parasites/μl down to 0.05 parasites/μl diluted in water containing gDNA from an uninfected donor (henceforth referred to as “infected standard”). *P. falciparum* (3D7) gDNA was isolated from parasite cultures with >4% parasitaemia at schizogony using the QIAmp DNA Mini kit. DNA concentrations were determined by spectrophotometry (Nanodrop Lite, Thermoscientific).

### Conventional PCR amplification followed by nested quantitative real-time PCR

Primer sequences and references are listed in Table 1. Initial rounds of amplifications were performed in 25 μl reactions containing 1 μl of template DNA, 0.5 μM of PLU5/PLU6 forward and reverse primers, and 1x KAPA2G Fast HS Ready Mix (Kapa Biosystems). In a conventional thermocycler, the template DNA was denatured at 95**°**C for 5 min, followed by 15 cycles of amplification (95**°**C for 30 s, 61**°**C for 30 s, and 72**°**C for 1 min) and a final extension at 72**°**C for 1 min. A reaction using 1 μl of PCR-grade water in lieu of template DNA was always included as a negative control in this first round of amplification.

**Table 1:** **Primers used for nucleic acid amplification**

Primer name	Sequence	Reference
rPLU5	5′ CCTGTTGTTGCCTTAAACTTC 3′	Snounou, G. *et al.* 1993 [12]
rPLU6	5′ TTAAAATTGTTGCAGTTAAAACG 3′	
rFAL1	5′ TTAAACTGGTTTGGGAAAACCAAATATATT 3′	
rFAL2	5′ ACACAATGAACTCAATCATGACTACCCGTC 3′	
Human GAPDH F2	5′ CGACCACTTTGTCAAGCTCA 3′	Smith, P.H. et al. 2011 [13]
Human GAPDH R2	5′ GGTGGTCCAGGGGTCTTACT 3′	

For the second round of amplification, 1 μl of the PCR product from the initial amplification was used as the template in a qPCR reaction (20 μl final volume) containing 0.2 μM FAL1/FAL2 forward and reverse primers and 1X Power SYBR Green Master Mix (Applied Biosystems). Reactions were performed in 384-well optical PCR plates on an Applied Biosystems 7900HT Fast Real-Time PCR system per the manufacturer’s recommended PCR parameters (50**°**C for 2 min, 95**°**C for 10 min followed by 40 cycles of amplification [melt at 95**°**C for 15 s, anneal/extend at 60**°**C for 1 min]) with the addition of a dissociation stage for subsequent melting curve analysis. In addition, 1 μl of serially diluted *P. falciparum* plasmid and infected standards were used as direct templates (i.e. without prior amplification) for qPCR reactions.

The nested qPCR protocol was originally tested using 10, 15, 20, 25, and 30 cycles for the initial amplification, with 15 cycles yielding the largest absolute difference in threshold cycle (Ct) values between the sample with the lowest and highest gDNA dilutions. Each experimental run included, as *Plasmodium* negative controls, a no template control (PCR-grade water as noted above) and gDNA extracted from donors previously determined to be *Plasmodium* negative by PCR, and, as positive controls, *P. falciparum* (3D7) gDNA, and the serially diluted, infected standard. All samples were run in duplicate or triplicate. As a DNA extraction control, real-time PCR was also performed on gDNA extracted from DBS using the human GAPDH primers in lieu of FAL1/FAL2 primers (Table 1).

### Quality assurance using external DBS samples

To validate the assay, the above protocol was run using gDNA extracted from DBS samples provided by an external laboratory and used in a recent study on quality assurance of *Plasmodium* PCR [14]. After determining calculated parasite densities using the protocol described herein, operators were un-blinded to the parasite densities calculated by the external laboratory, which employed a different qPCR protocol targeting the *P. falciparum* lactate dehydrogenase gene as previously described [15].

### Data analysis

ABI PRISM 7900 SDS software (version 2.4; Applied Biosystems) was used to evaluate the amplification and dissociation curves and determine the Ct values. Statistical analyses were performed in Prism 6.0 (GraphPad). Parasite densities (parasites/μl) and starting copy number of *P. falciparum* 18S rRNA plasmid from the standards were plotted against Ct values derived from the nested qPCR assay to generate standard curves. For all samples with “unknown” parasite concentrations, parasite densities were estimated from a regression line fit to the linear part of the standard curves using the average of nested qPCR-derived Ct values (performed in duplicate for all samples). The strength and significance of correlations were assessed with the Pearson’s correlation coefficient.

## Results

**Figure 1:**
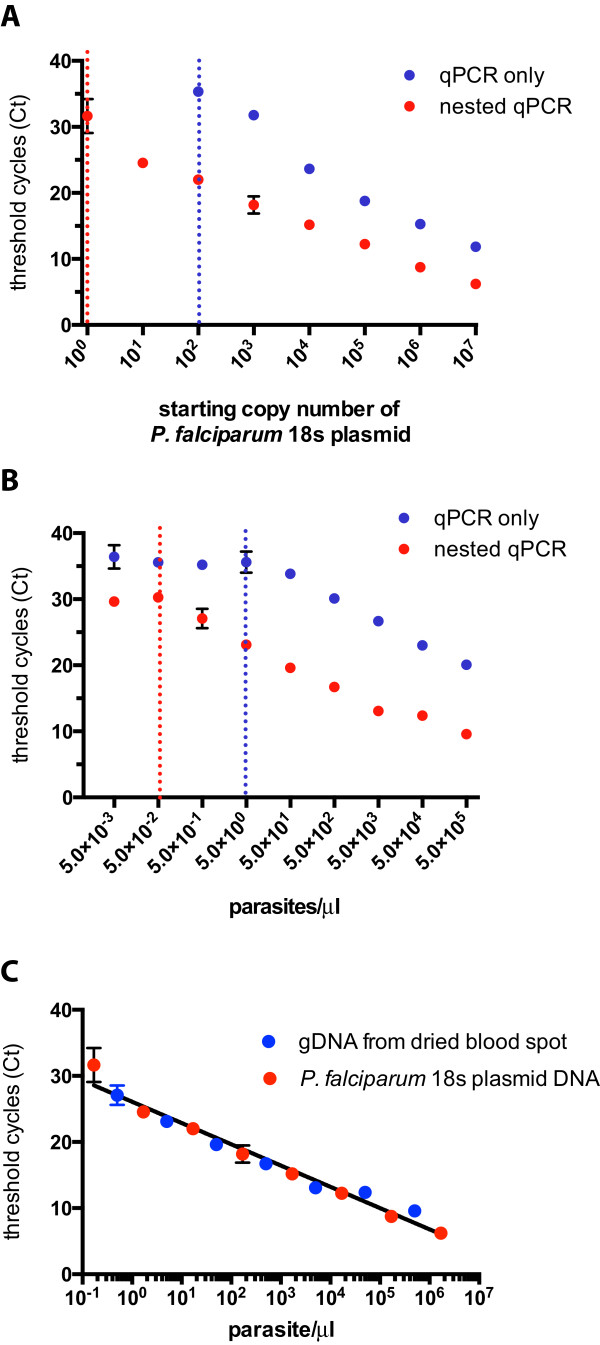
**Comparison of quantitative real-time PCR standard curves.** 10-fold plasmid or gDNA dilutions were plotted against Ct values generated from qPCR only and nested qPCR assays using **(A)** *P. falciparum* 18S rRNA plasmid or **(B)** gDNA extracted from dried blood spots obtained from a subject with clinical malaria as template DNA (described in the Methods). Points represent the mean of technical duplicates and error bars (where visible) indicate the standard deviation. Dotted lines represent the technical limit of detection as defined as the lowest template concentration for which there is a linear relationship between Ct values and copy number/parasite density. For **(C)**, Ct values generated by nested qPCR were plotted against the parasite densities estimated from copy number for *P. falciparum* 18S rRNA plasmid or corresponding to the dilutions from the clinical DBS standards. The best-fit regression line is shown as a black line.

**Figure 2:**
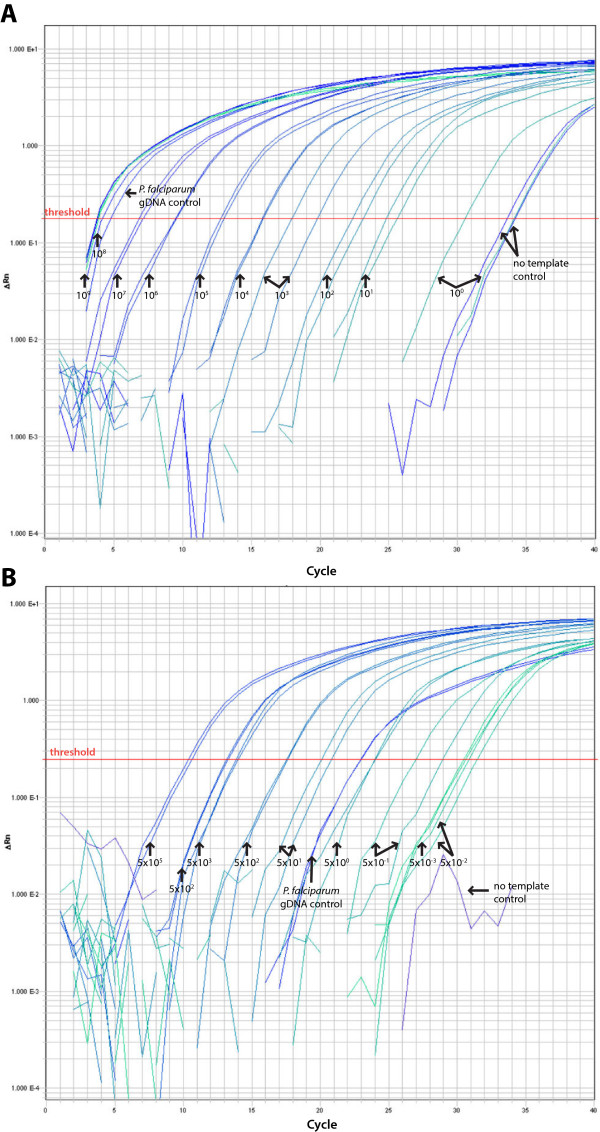
**Amplification curves for nested qPCR assay.** Nested qPCR amplification curves using 10-fold serial dilutions of **(A)** *P. falciparum* 18S rRNA plasmid or **(B)** gDNA extracted from dried blood spots obtained from a subject with clinical malaria as template DNA (described in the Methods).

Standard curves plotting Ct values against starting copy numbers or parasite densities (parasites/μl) were generated from serially diluted plasmid DNA containing the *P. falciparum* 18S rRNA gene or infected standard, respectively, for both 1) 15-cycle standard PCR amplification followed by nested qPCR and 2) amplification by qPCR only (Figure 1). Based on an estimate of 6 copies of 18S rRNA per *P. falciparum* genome [10], the limit of detection using *P. falciparum* 18S rRNA plasmid is 0.17 parasites/μl (equivalent to 1 copy/μl) for nested qPCR and 17 parasites/μl copies by qPCR only (equivalent to 100 copies/μl; Figures 1A and 2A). Similarly, the limit of detection using infected standards is 0.05 parasites/μl for nested qPCR and five parasites/μl for qPCR only (Figures 1B and 2B). To evaluate whether the two different sources of template DNA (*P. falciparum* 18S rRNA plasmid versus infected standard) yielded directly comparable parasite density estimates based on their Ct values, the Ct values generated by nested qPCR were plotted against the parasite densities estimated from copy number for *P. falciparum* 18S rRNA plasmid or corresponding to the dilutions in the case of infected standards (Figure 1C). A strong, negative correlation existed between parasite density and Ct values when values from both sources were combined (Pearson’s r −0.99; 95% CI [−1.0 to −0.96]; *P* < 0.001). High Ct values (>33 cycles) generated in samples with no template during the first amplification (Figure 2A) were confirmed as negative by inspection of dissociation curves (Additional file 1).

**Figure 3:**
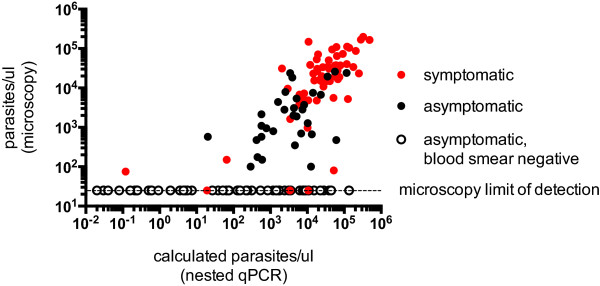
**Correlation between parasite densities determined by light microscopy and calculated by nested qPCR.** Calculated parasite densities from nested qPCR were plotted against parasite densities determined by light microscopy for symptomatic infections (red circles), asymptomatic infections with positive blood smears (black circles), and asymptomatic infections with negative blood smears (unfilled circles). Parasite densities calculated by nested PCR strongly correlated with both asymptomatic (Pearson’s r = 0.58, 95% CI [0.29 to 0.77], *P* < 0.001) and symptomatic (Pearson’s r 0.70, 95% CI [0.53 to 0.81], *P* < 0.0001) *P. falciparum* infections. The dashed line represents the limit of detection for blood-smear microscopy (~40 parasites/μl).

Parasite densities derived from nested qPCR were correlated to parasite densities determined by microscopy of concurrent blood smears for smear-positive patients who were either symptomatic or asymptomatic at the time of DBS collection (Figure 3). Parasite densities calculated by nested PCR correlated strongly with both asymptomatic (Pearson’s r = 0.58, 95% CI [0.29 to 0.77], P < 0.001) and symptomatic (Pearson’s r 0.70, 95% CI [0.53 to 0.81], P < 0.0001) *P. falciparum* infections. For asymptomatic patients who had negative blood smears but were positive by the non-quantitative nested PCR screen, the median parasite density derived from nested qPCR was 220 parasites/μl (interquartile range, 3.6-5100).

### Correlation of calculated parasite densities in independently validated *P. falciparum*DBS samples

Calculated parasite densities for 8 external DBS samples determined using our nested qPCR assay strongly correlated with values determined by an independent qPCR assay [15] (Pearson’s r 0.99, 95% CI [0.96 – 1.0], *P* < 0.0001).

## Discussion

Accurate quantification of low-density parasitemia from DBS samples has become increasingly important in clinical studies of malaria in which the kinetics of early or treated infection are approximated and for the identification of submicroscopic infections that serve as occult reservoirs for malaria transmission. In contrast to amplification protocols that quantify *Plasmodium* nucleic acid extracted from cryopreserved blood samples, which have a sensitivity of 0.02-0.06 parasites/μl [4], protocols employing direct qPCR of parasite gDNA extracted from clinical DBS samples typically cannot quantify samples with <5 parasites/μl [10, 14]. Nested qPCR, in which the product of the initial conventional PCR is used as the template for a second amplification with quantitative real-time PCR, has been used successfully to increase assay sensitivity in the diagnosis of tuberculous meningitis [16], respiratory viruses [17] and, more recently, *Plasmodium* infection [18].

In this report, a qPCR-only approach was directly compared to nested qPCR using standard curves generated with gDNA from actual clinical DBS samples as well as plasmid DNA. By incorporating a nested amplification with qPCR, the limit of detection was increased by about two orders of magnitude over a qPCR-only approach (Figure 1). However, a conservative estimate of the limit of quantification, and thus the practical sensitivity of the nested qPCR assay, is ~0.5 parasites/μl based on the increased variance at the lowest detectable dilutions and reproducibility with infected standard curves performed by multiple operators. This compares well with the sensitivity of ~2 parasites/μl in a recent study which also used a nested qPCR approach [18] with the small difference in sensitivity possibly attributable to the amount of whole blood initially used (5 μl versus ~10 μl from three 3-mm punches in this study) and differences in DNA extraction protocols, master mixes, and cycling parameters. Notably, the results here indicate that a *P. falciparum* 18S rRNA plasmid-based standard curve can be a reliable option in cases where high-parasitaemia clinical samples are not available, as parasite density estimates from Ct values are remarkably similar irrespective of whether the standards were prepared from plasmid DNA or gDNA extracted from a clinical DBS sample.

The importance of highly sensitive molecular diagnostics in clinical trials of malaria vaccine and therapeutic candidates and in campaigns to eliminate parasite reservoirs in endemic populations has placed an emphasis on proper standardization and quality assurance of nucleic acid testing protocols [4, 14]. The strong correlation between parasite densities calculated from the nested qPCR assay described herein with those from an independent protocol [15] provides confidence that the nested qPCR assay described here generates results that may be directly comparable to other studies using validated qPCR protocols. Furthermore, we also tested our assay over a wide range of parasitaemia from clinical DBS samples to evaluate its utility for field isolates. For patent infections, parasite densities derived from nested qPCR Ct values correlate well with those determined by microscopy both for symptomatic and asymptomatic cases (Figure 3). For submicroscopic infections, qPCR generally overestimates the parasite densities. One possible explanation is that thick blood smears generally underestimate the true parasite density due to parasite loss during staining [19] and, in the case of the highly discordant samples, the loss may have been particularly marked. Another explanation may be reduced precision between replicates due to small volumes of template used in the reactions. One microliter of PCR product was used as the template for the second amplification to maximize the dynamic range of the assay, which allowed quantification of either submicroscopic or high-density parasitaemia using a single protocol. However, if only submicroscopic or low-density parasitaemia is being considered, increasing the template volume for the second amplification may improve not only the precision, but also the sensitivity of the assay. Conversely, on occasion nested qPCR underestimated the parasite density relative to microscopy, but much less frequently (Figure 3). This phenomenon might be explained by the presence of human gDNA interfering with amplification of the parasite gene rather than inefficient DNA extraction, given that appropriate extraction was verified by the presence of human GAPDH.

A major limitation with the nested qPCR approach is the increased chance of false positives given the two cycles of amplification. Thus, care must be taken to reduce the possibility of false positives due to contamination. Of note, amplification curves were occasionally observed to have Ct values of >32 cycles for the negative (no template) control (Figure 2A), but these consistently appear to be primer dimers by dissociation curve analysis (Additional file 1).

Based on these findings, it is recommended that a negative control reaction always be included with the first amplification and that any Ct value within 2 cycles of the negative control be considered negative with additional confirmation by dissociation curve analysis. One way to minimize false positives is to always employ an initial screen for *Plasmodium* infection using a conventional nested PCR approach (based on the original assay described by Snounou *et al.* [12]), and selecting only confirmed *P. falciparum*-positive samples for subsequent quantification with nested qPCR as done in this study. This two-step screening approach, although more time-intensive, would also minimize expending qPCR reagents on uninfected samples in malaria surveillance studies.

The nested qPCR approach in this study may be particularly convenient in laboratories that already employ the standard nested PCR protocol developed by Snounou *et al.* [12] given that the same primer sets are shared between the protocols. Although this assay was only validated for the quantification of *P. falciparum* parasitemia, applying a nested qPCR approach to the other four human *Plasmodium* species would be relatively straightforward if species-specific *Plasmodium* 18S rRNA primers were used during the real-time amplification, as was done in a recent study [18].

## Conclusions

The nested qPCR assay presented here reliably quantifies parasite densities from DBS samples obtained from subjects with asymptomatic and symptomatic malaria and achieves higher sensitivity than a qPCR-only approach. This assay may be useful for active malaria surveillance in areas where submicroscopic asymptomatic infections are prevalent.

## Electronic supplementary material

Additional file 1: **Nested qPCR dissociation curve for no template negative control and** ***P. falciparum*** **gDNA positive control.** (PDF 427 KB)
