# Supplementary figures and images for: A nested real-time PCR assay for the quantification of Plasmodium falciparum DNA extracted from dried blood spots
Source: Malar J. 2014 Oct 4;13:393. doi: 10.1186/1475-2875-13-393 (PMC4197274; doi:10.1186/1475-2875-13-393)

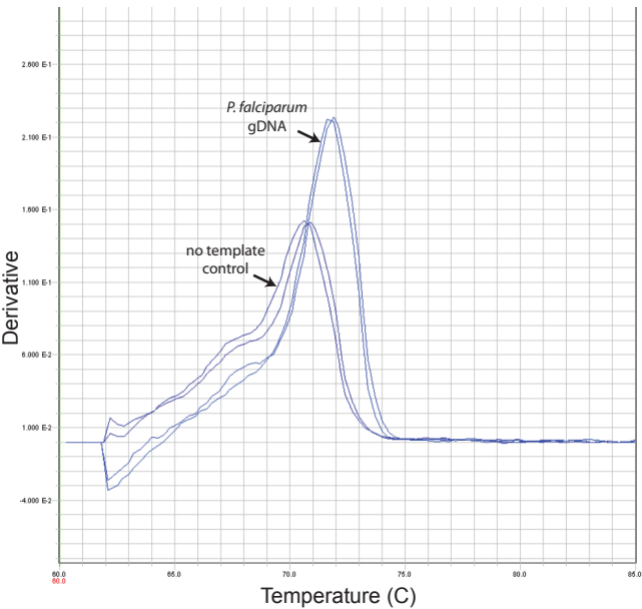

Supplement: Supplementary file 1 — Additional file 1: Nested qPCR dissociation curve for no template negative control and P. falciparum gDNA positive control. (PDF 427 KB) [file 12936_2014_3556_MOESM1_ESM.pdf]
